# The augmented radiologist: artificial intelligence in the practice of radiology

**DOI:** 10.1007/s00247-021-05177-7

**Published:** 2021-10-19

**Authors:** Erich Sorantin, Michael G. Grasser, Ariane Hemmelmayr, Sebastian Tschauner, Franko Hrzic, Veronika Weiss, Jana Lacekova, Andreas Holzinger

**Affiliations:** 1 Division of Pediatric Radiology, Department of Radiology, Medical University Graz, Auenbruggerplatz 36, A – 8036 Graz, Austria; 2 Faculty of Engineering, Department of Computer Engineering, University of Rijeka, Vukovarska 58, Rijeka, 51000 Croatia; 3 Institute for Medical Informatics, Statistics and Documentation, Medical University Graz, Graz, Austria

**Keywords:** Artificial intelligence, Clinical decision-making, Deep learning, Pediatric radiology, Radiomics

## Abstract

In medicine, particularly in radiology, there are great expectations in artificial intelligence (AI), which can “see” more than human radiologists in regard to, for example, tumor size, shape, morphology, texture and kinetics — thus enabling better care by earlier detection or more precise reports. Another point is that AI can handle large data sets in high-dimensional spaces. But it should not be forgotten that AI is only as good as the training samples available, which should ideally be numerous enough to cover all variants. On the other hand, the main feature of human intelligence is content knowledge and the ability to find near-optimal solutions. The purpose of this paper is to review the current complexity of radiology working places, to describe their advantages and shortcomings. Further, we give an AI overview of the different types and features as used so far. We also touch on the differences between AI and human intelligence in problem-solving. We present a new AI type, labeled “explainable AI,” which should enable a balance/cooperation between AI and human intelligence — thus bringing both worlds in compliance with legal requirements. For support of (pediatric) radiologists, we propose the creation of an AI assistant that augments radiologists and keeps their brain free for generic tasks.

## Introduction

Imaging is an integral part of medical diagnostics. Image acquisition is achieved by exploiting sophisticated technology, but image interpretation is still a task for the “human radiologist” [1]. To fulfill this complex task, people require almost three decades of learning as well as continuing medical education (CME) [2]. Despite all these efforts, perception and diagnostic errors exist.

Maturing imaging technology not only leads to steadily increasing temporal, geometric and radiometric resolution but also to new modalities — thus leading to an increasing number of images per case. As an example, a CT study for a trauma patient can consist of about 1,000 images. Additionally, imaging modalities have different, non-standardized interfaces.

**Fig. 1:**
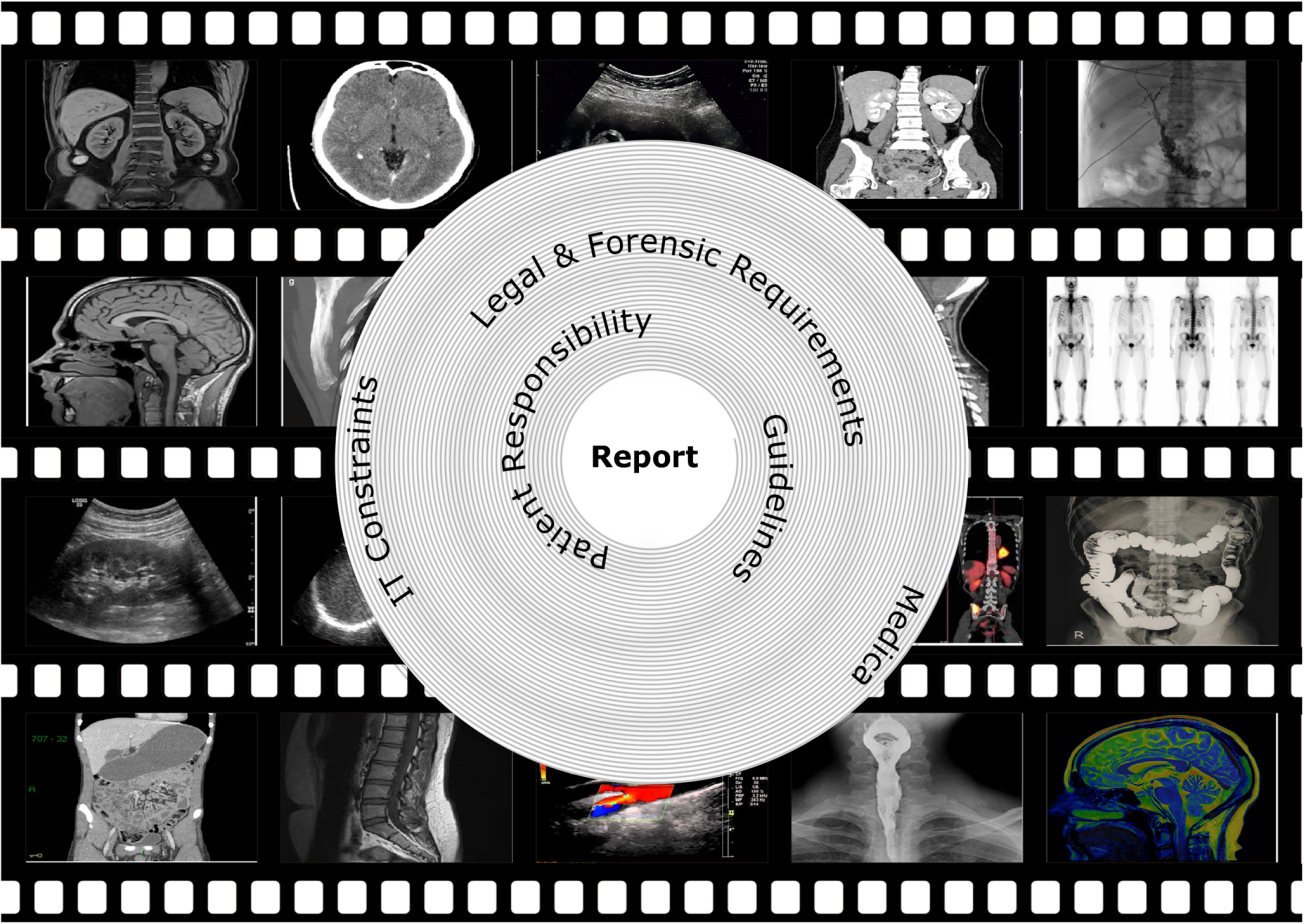
Visual representation of the radiologist’s workplace (cockpit), where images pass by like a film strip. Analyzing them can be envisioned as a tunnel, where the report is the final product. On the way to the report several constraints like patient responsibility, and legal and forensic requirements have to be handled

Radiologists’ workplace resembles a cockpit with streaming data and involves the management of several interfaces and information technology (IT) systems (Fig. 1). Despite their obvious advantages, all systems have constraints, inherited assumptions of how they should be used, thus handicapping the free flow of radiologists’ intellectual properties in report generation. There is the light on the firmament, that artificial intelligence (AI) will influence almost everything in medicine and will improve patient outcome in many ways. In regard to radiology, it is expected that radiologists’ reports will get better and more precise. Some researchers have even proclaimed the funeral of radiologists because AI will do it all [3]. Choy et al. [4] studied job prospects for radiologists with regard to the use of AI. They concluded that in the foreseeable future there will be no reduced need for radiologists [4]. A recent PubMed search with the keywords “artificial intelligence” and “radiology” yielded 5,641 papers since 1986. To put the focus on pediatric radiology, we applied the PubMed age filter “child: birth–18 years,” thus narrowing the search to 308 papers (5.4%). Only a small proportion was genuine research in pediatric radiology: Miyagawa et al. [5] predicted suspected increased intracranial pressure by using machine learning in children; other recent pediatric publications dealt with deep-learning techniques for pulmonary-thoracic segmentation [6] and measurements of leg-length discrepancy [7], to name a few. In other publications, children or adolescents were included with adults.

Therefore, the purpose of this paper is to describe the current (pediatric) radiologist’s environment — including IT support, its inherent shortcomings as well as current AI applications and their difficulties. In addition, we present a new approach called “explainable AI” and describe how AI can augment the pediatric radiologist.

## Radiologists’ workplace and report generation

**Fig. 2:**
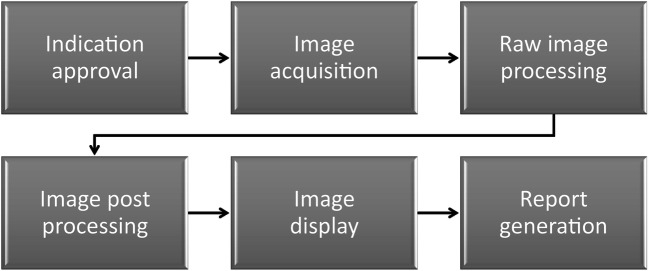
Imaging chain — simplified radiology workflow

Basically, radiology workflow consists of the steps as displayed in Fig. 2 [8, 9]. Typically, imaging studies are performed in teamwork with radiographers, where either imaging data are acquired by technologist alone (e.g., CT, MRI) or radiologists perform imaging studies by themselves (e.g., US, fluoroscopy, interventional radiology — depending on national legal environment). As mentioned, user interfaces of imaging modalities are not standardized and can involve all extremities. As an example, feet can be used for execution of a “snapshot/video” on US machines or “fluoroscopy on/off” on C-arm systems [10]. During image analysis, a three-dimensional (3-D) model of patho-anatomy has to be kept in mind [11].

**Fig. 3:**
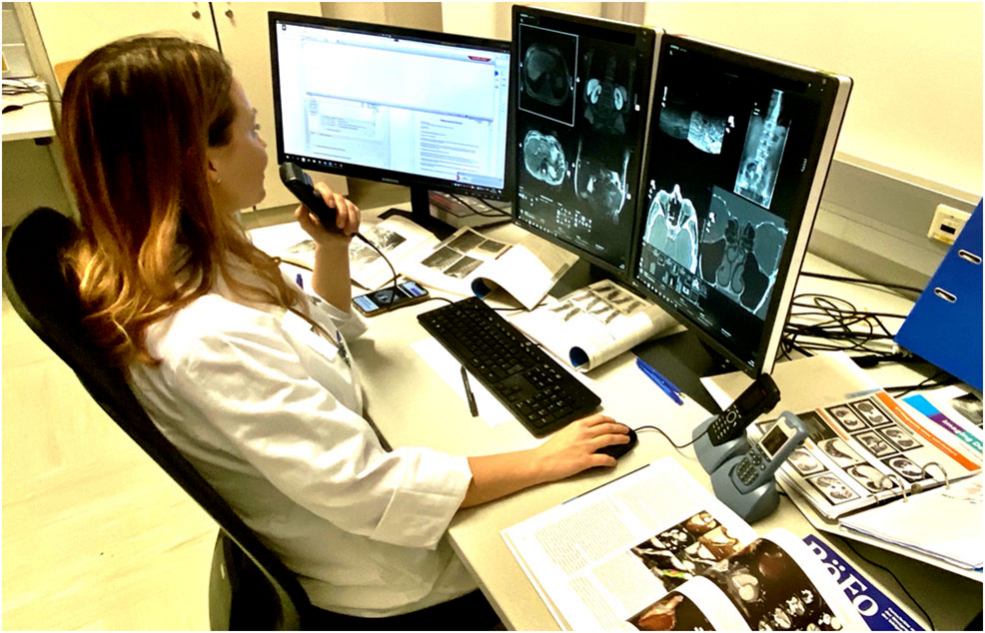
Photograph of typical radiologist’s workplace — high-resolution monitors, office monitors, keyboard, mouse, communication tools (landlines and mobiles), books, journals and memo sheets

Report generation is done in front of multiple monitors; some of them are licensed for image interpretation, while office monitors are used for more general purposes, e.g., for interaction with the hospital’s information system (HIS) or for assessing other sources of information like medical databases. Additionally, communication tools like landlines or mobiles as well as numerous textbooks and atlases on the bookshelf complete the picture. Figure 3 illustrates a typical workplace of a radiologist, including IT equipment and literature.

In the center of all this equipment and tools is the radiologist — he or she is managing data waves, trying to keep the shuttle on track and generate a smart report.

Moreover, findings of previous multi-modal studies as well as patients’ medical history and clinical signs and symptoms must be considered before diseases can be diagnosed — thus there is an increasing workload over the last years [12–14]. Additionally, equivocal findings must be clarified by checking diagnostic pathways in books, publications, pictorial essays or medical databases or by simply searching the internet for similar images.

European Union (EU) directives cover several aspects of work organization and determine permissible work assignment, and some of these apply to radiologists, too [15]. Within EU countries, extended and overnight shifts for radiologists, as for other medical staff, can last up to 25 h [16]. But still, there are no regulations on how these 25 h are spent, so there is an inherent assumption that radiologists will do the job as required.

To minimize errors in interpretation, strategies like double-reading were introduced. In highly sensitive areas like breast imaging, this technique increases sensitivity by 5–10% [17]. According to the European guidelines for quality assurance in breast cancer screening and diagnosis, a predefined protocol for quality control exists to minimize errors [18]. In pediatric radiology, such regulations do not exist. Professional societies like the European Society of Paediatric Radiology publish guidelines through task forces [19]. Within these professional societies, it is common to share troublesome, non-urgent cases with specialists abroad or discuss them at scientific meetings.

### Information technology support and shortcomings

Current IT support consists of several parts. Picture archiving and communication systems (PACS) free radiology departments from managing analog film archives — film archiving and retrieval is no longer necessary. Patients can be managed almost paperless by HIS, which provides patients’ history, images from previous examinations, reports and more.

Soft-copy reading is augmented by hanging protocols. Depending on the imaging modalities used, the visualization software can be configured to display images in the best way for reading, thus freeing the radiologist to shuffle them around. Therefore, information like modality, body part, study description, image orientation and patient positioning or current procedural terminology (CPT) codes must be available for each study [20]. Hanging protocols can be stored centrally and thus made available on different workstations so they can be used by different users/groups. To enable sharing, standards have been developed in terms of a “hanging protocol service class” and a “hanging protocol composite information object definition” [21].

Today software is available, running in the background/cloud, that highlights pathology and supports the radiologist in diagnosing, for example, brain disorders, pulmonary embolism and more [22]. Powerful workstations permit 3-D reconstructions and other post-processing tasks almost in real time. Speech recognition enables direct transcription of the spoken word to text for a report.

Customization of these systems to the department’s workflow can be an endless task — starting with catalog generation, prefetching rules, definition and consent finding for hanging protocols, and more. Everything must be organized in compliance with applicable data protection law. Software interfaces for post-processing workstations are not standardized and are sometimes not even from the same vendor.

Cloud computing is becoming popular, and cloud-based AI applications exist. An overview and information about type of clearance, either CE (Conformité Européenne) or FDA (United States Food and Drug Administration) is available on the internet [23]. It has to be kept in mind that the European CE label refers only to formal law such as data protection or security issues; it does not give any information about the AI algorithm performance. To enhance transparency and performance comparability among AI algorithms, the American College of Radiology (ACR) offers a testing opportunity on appropriate reference standard data sets, collected from multiple institutions, thus representing ground truth [24]. Moreover, integration of tools like speech recognition offers other challenges. After individual software training, in daily use radiologists have to open new windows on screens and wait for synchronization of the involved speech recognition, e.g., with HIS. Speech recognition depends on the thesaurus used and the statistical representation of words. There are dedicated thesauruses for radiology but these are usually not developed for pediatric radiology. Therefore, transcription rates for chest films, CT and MRI are good and reach the desired performance level of 95%, but according to the authors’ experience, these rates are lower for US, voiding cystourethrography, video-urodynamics and defaecography, for example [25, 26]. Additionally, reports with numerous numerical data like cardiac CT or MRI suffer from less recognition. Only a few systems allow integration of forms or switching between text and numerical input.

Overall, it is always a balance between individuality and standardization. Mutual understanding of engineers and radiologists is a key feature but not always present.

### Structure of reports and structured reports

The final report is the result of reading a study. It is a formal document that obliges the radiologist to give an official interpretation of an examination or procedure [27]. For decades there have been guidelines about structure and wording from Wang et al. [28], Wallis and McCoubrie [27] and the European Society of Radiology (ESR) [29]. An ACR inter-society conference concluded that reporting tools should not impede the productivity of radiologists. Reporting tools should be able to integrate speech recognition and structured reporting for radiologists [28]. In addition, radiology organizations should create a report repository based on standard vocabulary [28]. Standards like the Clinical Document Architecture (CDA) and the Digital Imaging and Communications in Medicine (DICOM) part 20 for translation of DICOM structured reports into CDA documents enable integration within an IT environment [29]. There is no question about the importance and usefulness of these guidelines, but it is still an open question how much brainwork radiologists are using for the formal requirements versus the medical question and diagnostic puzzle. This applies in particular to residents, where IT handling might be easier to Generation Y but where the main focus is to acquire knowledge and skills in radiology. On the contrary, the Baby Boomer generation struggles more with IT system management, thus handicapping their workflow.

***This leads the authors to STATEMENT ONE: Managing the radiologic IT environment represents a complex task that consumes inappropriate mental power. It is our hope that radiologists will be supported soon by intuitive and seamless AI applications***.

But what is the solution? Better, more fail-proof, simpler and more usable systems? Or assisting radiologists with a new kind of AI assistant, where radiologists concentrate on medical content and work closely/jointly with an AI expert in one workplace? Who will take on the costs when rather the opposite is desired: replacing the radiologist with AI to save costs?

## Artificial intelligence

The idea of AI was first published in 1950 by Alan Turing [30] when he hypothesized in his paper: “Can computers reason as well as humans?” This was the starting point for many definitions, but from a computer science perspective, it could be stated that “AI represents the ability of a digital computer or computer-controlled robot to perform tasks commonly associated with intelligent beings” [31, 32]. Computers must derive their decisions/conclusions from patterns/policies in order to accomplish those tasks. Methods used are referred to as machine learning methods or machine learning algorithms.

Machine learning uses computer algorithms to improve automatically through experience. All algorithms share three fundamental properties: (a) data analysis [33], (b) model and model optimization (machine learning algorithms or methods are usually referenced as the model) [34] and (c) goal function or cost function (term used for a function measuring the performance of the model on a given task) [35].

**Fig. 4:**
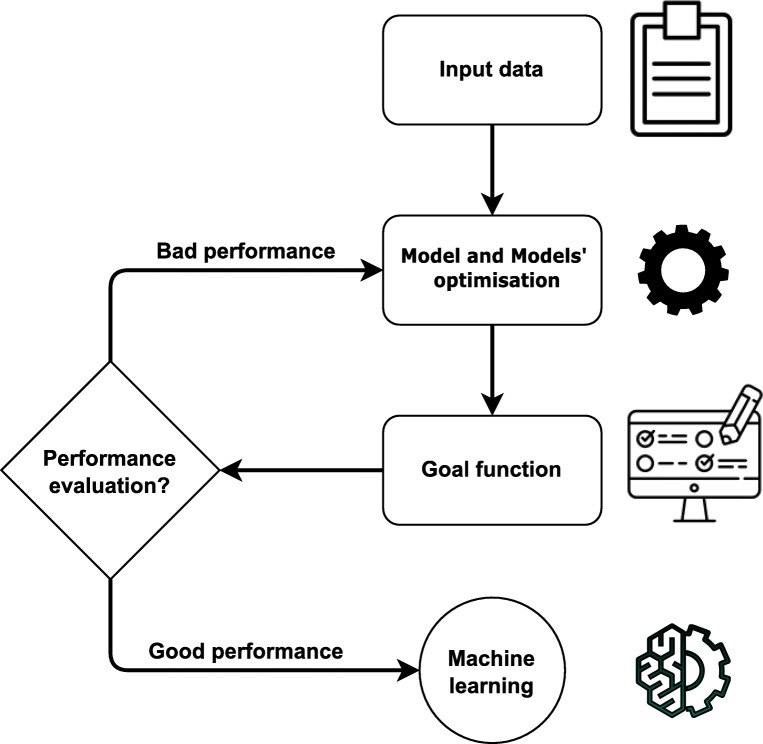
Life cycle of a machine learning method. The first phase is obtaining the data, followed by choosing and optimizing a model. The last step is developing a final model with good performance that can be used in practice

These three parts represent a life cycle of a machine learning model, which is presented in Fig. 4. The four basic machine learning categories are based on the problem and data: (1) supervised learning, or classification and regression of problems where data are labeled [36]; (2) unsupervised learning, or clustering and grouping of unlabeled data [37]; (3) semi-supervised learning, where unsupervised methods help supervised methods to increase accuracy [38, 39]; and (4) reinforcement learning, also known as learning through trials, where AI learns how to control an agent in the dynamic world [40]. Machine learning methods are often confused with statistical methods/models. Machine learning is all about results and conclusions, whereas statistical modeling and statistical methods are more about finding relationships and the significance of the relationships between variables.

### Deep learning

Deep learning refers to a subset of machine learning methods based on algorithmic interpretation of neural networks as found in any living being [41]. In 2006, G. Hinton et al. [42] made a huge impact on neural networks training and availability by learning a high-level representation based on successive layers of binary or real-value latent variables with a restricted Boltzmann machine. As a result of their research, several types of neural networks have been created:

*Feedforward neural networks* represent the simplest form of neural network, where information moves in only one direction [43]. *Convolutional neural networks* are based on two-dimensional (2-D) convolution operation and are typically used to extract information from images [44]. *Recurrent neural networks* are like feedforward neural networks except recurrent neural networks allow cycles within the hidden layers. Cycles serve as memory cells that can store important features in causal data types [45].

**Fig. 5:**
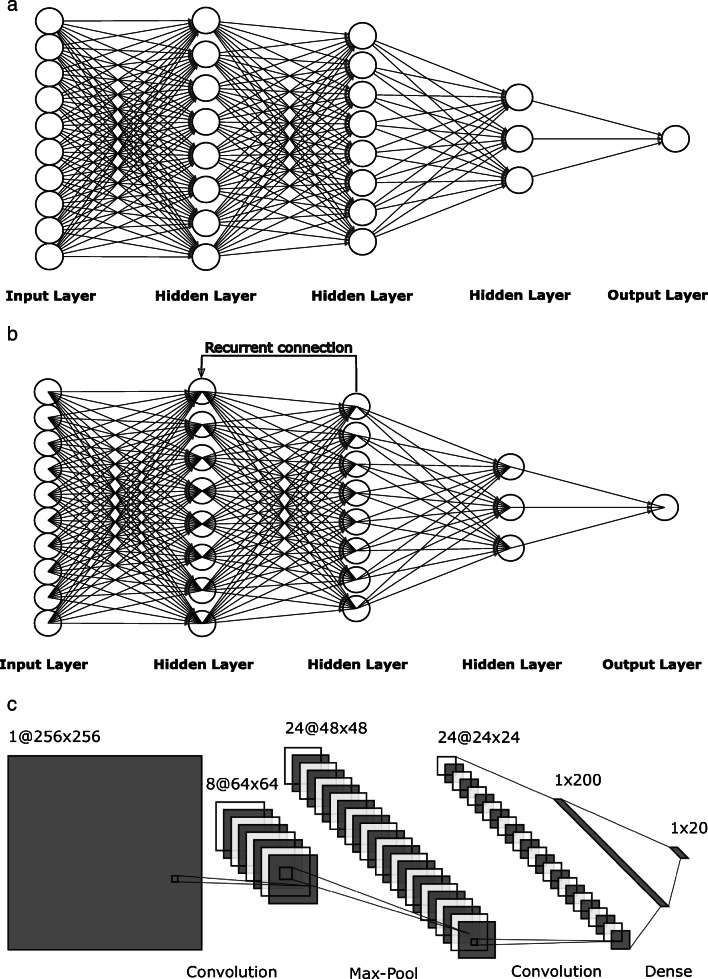
Examples of neural network topology schemes. **a** Feedforward neural network topology. **b** A typical recurrent neural network topology. **c** A convolutional neural network topology

**Fig. 6:**
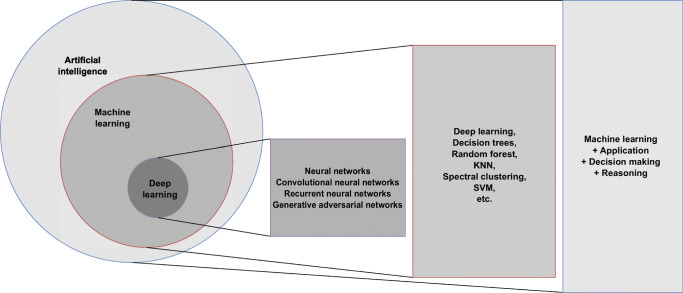
Overview of the relationship among artificial intelligence (AI), machine learning and deep machine learning. Machine learning is a subtype of AI, and deep machine learning is a subtype of machine learning. Examples of methods are given for each subtype. *KNN* K nearest neighbor algorithm, *SVM* support vector machine algorithm

The common topology representations for each of the neural network types are given in Fig. 5. To summarize, deep learning is a part of machine learning that makes artificial intelligence possible [46]. The overview of the relationship among AI, machine learning and deep machine learning is presented in Fig. 6.

### Artificial intelligence applications in medicine

**Fig. 7:**
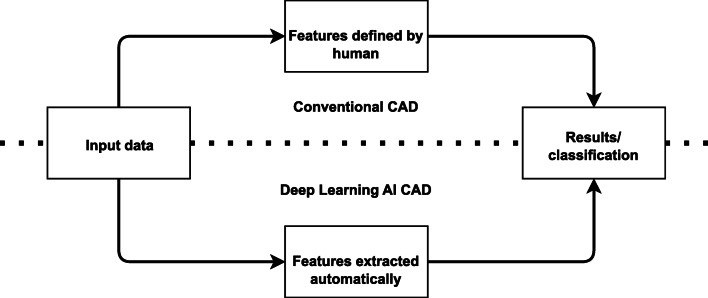
Differences between conventional computer-aided diagnosis (CAD) and deep learning artificial intelligence (AI) CAD systems

Radiomics is a method that extracts a large number of features from images and can help set up diagnosis [47]. The general idea is to extract several image features such as size and shapes of different regions, descriptors of the image intensity histograms, different texture extractions or irregularities, etc., that can be helpful in diagnosis, predicting prognosis, and therapy management for various conditions [48, 49] These features form the basis for computer-aided diagnosis (CAD) software [50]. The leading purpose of CAD software is to reduce human labor and increase efficiency. CAD, as shown in Fig. 7, can be divided into two types based on how features are extracted: conventional CAD, with features proposed by humans; and deep learning AI CAD, where useful features are proposed and learned by machine learning [51].

Before the introduction of neural networks, and thus AI, scientists used machine learning methods such as regression, hand-made pattern recognition and support vector machines (SVM)-based approaches to detect anomalies or extract diagnostic patterns [52–55]. As AI evolved, features extracted by the algorithms became more progressive [56]. In some cases it was shown that human performance was achieved or even surpassed [41, 57]. For instance, it became evident that AI can detect and characterize certain cancers by distinguishing benign from malignant nodules that are not visible to the human eye [58]. Given the better cure rate of early stages, this could greatly improve patient outcomes. Also, through automatic segmentation, AI offers enormous potential in terms of efficiency, reproducibility and quality of measurements [59]. However, for proper AI performance, a huge amount of high-quality data is necessary, which poses a major challenge [60].

Other AI applications in radiology are targeted to speed image acquisition, lower radiation exposure [61, 62] and improve image quality [63–65] or speech recognition to support transcription [66].

### Challenges and shortcomings of applied artificial intelligence in medicine

Because of the success of machine learning, particularly deep learning [40], AI is experiencing an enormous renaissance, and successful radiology examples have been mentioned [67]; however, there are new challenges [68]. Considering AI systems as black boxes represents a major problem in terms of traceability and thus explainability.

Increasing legal demands for explainability, particularly in the European Union [69], raise the question of *why* a result has been achieved, which is not only desirable but mandatory! For radiologists, this requires technical possibilities to be able, on demand, to retrace, understand and interpret *how* results were obtained by AI [70]. Consequently, a growing community is working in the field called explainable AI (xAI) to develop methods to make such black box approaches interpretable by humans.

**Fig. 8:**
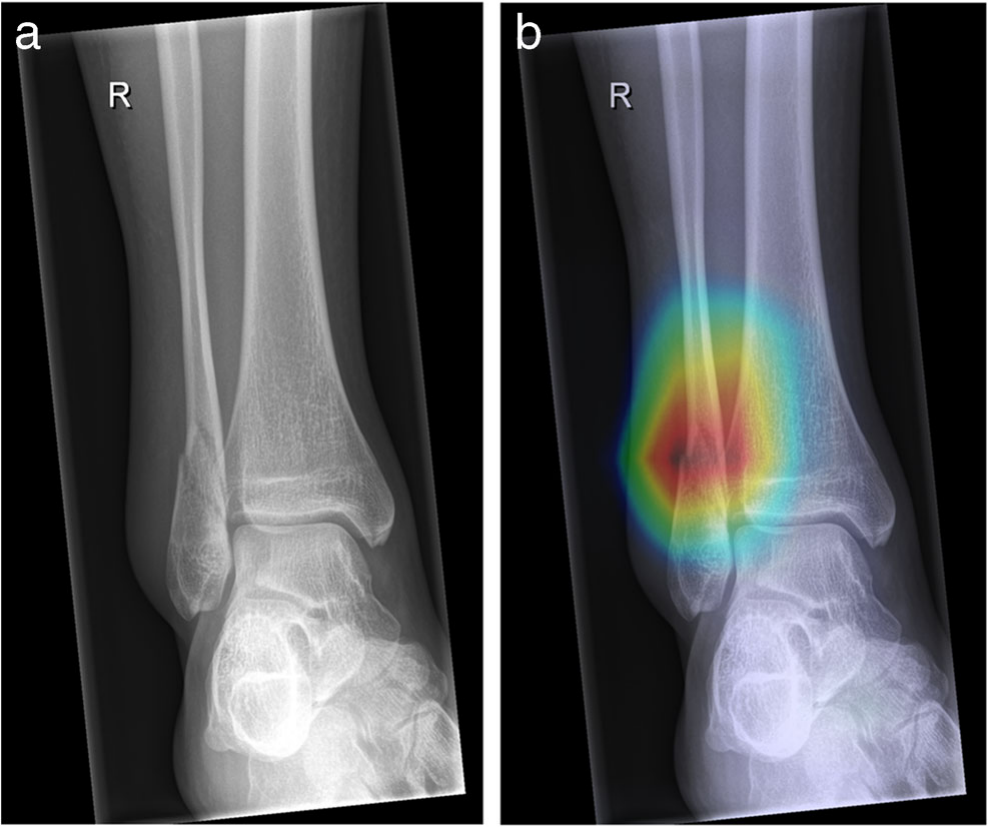
Layer-wise Relevance Propagation in a 15-year-old girl. **a** Anteroposterior (AP) ankle radiograph shows a fibula fracture. **b** Radiograph was sent to an artificial intelligence (AI) model trained for automated fracture detection. AI-predicted pathology is depicted as a heatmap overlaying the radiograph, where red (hot) areas represent regions where AI is more confident of a fracture. Heatmaps are especially useful when checking model predictions in regard to accurateness and plausibility

A popular example of such an explainable AI method is Layer-wise Relevance Propagation [71], wherein relevant parts of the input data that caused a result can be highlighted utilizing heatmaps. An example is presented in Fig. 8, where more red areas represent a higher likelihood of fractures.

This technical solution is an excellent first step: explainability highlights decision-relevant parts of machine re-presentations and machine models, i.e. parts that contributed to model accuracy in training, or to the confidence of a prediction. In the medical domain there is need for the concept of causability [72]. Causability is the measurable amount of an explanation — in the technical form of a heatmap, as shown in Fig. 8 — achieving a specified level of causal understanding for humans [73].

The word “causability” comes from “usability” because usability encompasses measurements for the quality of use and has been accepted in software engineering for a long time [74]. This approach is very important in the medical domain generally and in radiology specifically. For legal reasons, humans remain in control and are responsible for decision-making — even when AI is doing/supporting the decision.

A further reason why fully automated AI will not prevail in the mid term is represented by the fact that physicians/radiologists own a conceptual understanding and experience that no AI has to date. A potential solution can be interactive machine learning with the “human in the loop” [75].

The amount and quality of data needed for machine learning training are important factors for its success. In detail, the following facts have to be mentioned. Regarding *data quality*, frequently signal-to-noise ratio is used as a metric. The noise and presence of the artifacts depend on motion blur, proper handling of the equipment as well as the equipment settings [76]. Regarding *dimensionality*, imaging data can be 2-D images up to 3-D or 4-D volumes with high-resolution and complex structures, but machine learning algorithms work with small image sizes (typically matrices have a size of 224×224). Down-scaling the input data to the required matrix size results in blurring or even loss of important details. Therefore, techniques were developed to keep only the important structures/parts even after a reduction of dimensionality [77].

*Data variability* requires complete calibration of imaging modalities, which is not possible in daily routine and is usually not needed clinically. As an example, for the same object there is a variation of attenuation coefficients by different CT machines, further varied by different exposure settings and image reconstruction techniques [78]. These differences add unnecessary complexity to the data sets that needs to be compensated by normalizing the data or performing different data augmentation techniques.

Some of these problems are easy to solve, but some are nearly impossible. However, as more data sets are becoming available to the public, there will be more research in algorithms and approaches to obtain invariant data [79–81].

## Humans vs. artificial intelligence in problem-solving

For possible future medical AI applications in general, and in pediatric radiology in particular, a very important question is this: What can be better done by AI and what can be better done by the (human) radiologist? A reasonable assumption is a combination of both — a step toward the “augmented radiologist.” The ultimate responsibility will be still the radiologist’s. An imaginable scenario would be a change of work quality: simple but time-consuming work would be left to the machine and complex work would still require human expertise. Extrapolation tasks should serve as a practical example. Making predictions from few or uncertain data is generally hard to achieve by machines [82]. This problem is even harder in the medical domain, which is full of uncertainty, and where there are rarely exact function values without any errors or noise [83]. Trained AI models can measure the similarity of two data objects; however, they cannot explain *why* they are similar. Here again, a human-in-the-loop can be of help to find the underlying explanatory factors of *why* two objects are similar because of his or her contextual understanding of the target domain, which is a typical feature of humans (radiologists).

As stated in the very beginning, a *true intelligent* learning algorithm can automatically learn from data, extract knowledge and make decisions similar to those of humans. Therefore, at all times AI has been inspired by humans, *how* they learn, how they extract knowledge and how they make decisions. Key insights from past research provide probabilistic modeling and neural-inspired algorithms [84]. Function learning appears in everyday cognitive activities: nearly every task requires *mental representations*, which map inputs X to outputs Y, *f*: *X* → *Y*. Because the set of such mappings is infinite, inductive biases need to constrain plausible inferences. Theories on how humans learn such mappings with continuous variables have focused on two alternatives: (1) humans are just estimating explicit functions, or (2) humans are performing associative learning supported by similarity principles. The group around Tom Griffiths at Princeton developed a model that unifies both these assumptions [85].

Studies are evidencing that humans are excellent at finding near-optimal solutions to difficult problems; they can detect and exploit some structural properties of the instance in order to enhance solution parts. It is interesting that medical doctors are not aware how hard and expensive it would be to solve these problems with AI [86, 87].

***This leads the authors to STATEMENT TWO: AI needs legal safety, security and accountability.***

As a result of legal and forensic constraints, it is not an option to use AI systems as black boxes — press a button and wait for the results, and either accept the AI suggestion or not. The reporting radiologist must be able to retrace and to ask questions of the system as to why a certain decision has been reached. This calls for an explainable AI with features like retraceability and interpretability. Future human AI interfaces should enable such a multi-modal view [88, 89].

## Personal experiences

**Fig. 9:**
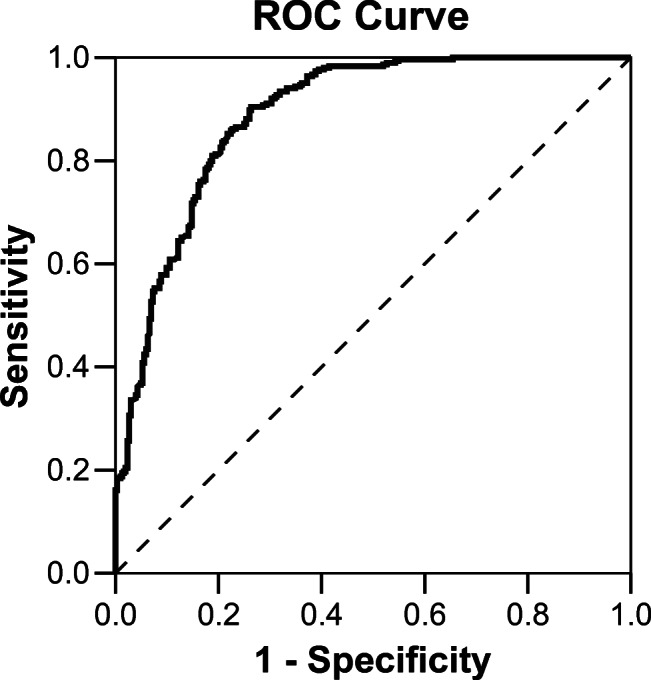
Receiver operating characteristic (ROC) curve of pediatric fracture classification. Area under the curve (AUC) is 0.889 with a 95% confidence interval (CI) of 0.863–0.915. The model was trained on 258,866 pediatric radiographs of all body regions

Our interdisciplinary research group started working in the field of pediatric trauma computer vision applications in 2018 [52]. As described [90, 91], AI can be helpful in the domain of automated fracture detection. One of the main hurdles in establishing AI algorithms is the lack of annotated training data sets and data quality. It is laborious work to create and maintain large image collections with proper labels [92]. We found AI algorithms to exceed pediatric radiologists regarding fracture detection in specific regions like wrist radiographs (Janisch et al., 2021, unpublished). We think that AI can achieve similar performance in most body regions and tasks, given enough training data. The auxiliary displayed heat map gives the reporting radiologist the possibility to analyze the “hot region” found by the AI system as well as the level of confidence (Fig. 8). Figure 9 shows a receiver operating characteristic (ROC) analysis of a general pediatric fracture classification algorithm trained on more than 200,000 radiographs in anteroposterior (AP) view of all body regions.

The results are not entirely satisfactory yet, given an accuracy of nearly 90%. However, this algorithm is based on the radiology reports only, which are positive or negative for a single study, but not individual images. Thus, an image might be labeled positive for a present fracture, even if it is not actually visible and perceptible to a human expert. Also, labeling errors need to be considered, depending on the experience of the labeling radiologist. Accuracy might be significantly improved by minimizing the present labeling issues and by developing region-specific models. Heat maps allow the reporting radiologist to get information about the AI system’s confidence level.

## How can artificial intelligence help (pediatric) radiology?

The daily workflow within radiology offers many options to include AI. One general application would be re-engineering the human–computer interface. For decades, a keyboard and mouse have been the main tools. Only smartphones, tablets and some laptop touchscreens allow for the use of fingers in a more intuitive way. Another approach would be to use gestures or spoken commands for interaction with IT systems. AI should work in the background and notify users by hovering notes on the corresponding HIS sections. A full HIS integration will be the cornerstone for acceptance by medical staff — it is not an option for them to spend their time serving several IT systems (see STATEMENT ONE).

Next, we discuss potential AI support according to the individual steps of the simplified imaging chain (Fig. 2).

### Indication approval

Referring physicians could get AI support for selecting the appropriate imaging procedure. Current clinical decision support systems are monolithic, thus allowing selection of one item [93]. An AI system could sum up the available patient information to suggest the best suited diagnostic pathway for a particular case.

### Image acquisition and raw image processing

A (stereoscopic) camera system and a weight sensor could automatically measure body dimensions and weight. By sharing this information with imaging devices, tight shutter settings could be augmented. In addition, in plain radiographs and CTs, appropriate exposure parameters as well as raw image reconstruction items (e.g., looking up tables, sharpening and more) could be suggested in a non-linear way. Measured patient weight could allow AI to calculate the amount of intravenous contrast medium needed for CT; these data could be transferred automatically to the power injector. The operator would be informed by the hovering notes. This would be a smooth process and radiographers could concentrate better on the (pediatric) patients instead of the machines. As mentioned, there are efforts to reduce either the dose in CT or speed up MRI by AI [61, 62, 64, 65]. The seamless integration of deep learning in CT image reconstruction has already been achieved by one vendor [94, 95]. These data could be exploited further by linking them with parameters of image quality — thus forming the basis for a periodic report that could represent a valuable future tool in quality management.

### Image post-processing

Image post-processing tasks such as image segmentation, multidimensional image reconstructions, or generation of parameter images (displaying function, e.g., perfusion maps instead of anatomy/pathology) could be greatly enhanced by AI. Today’s image segmentation tools are crude, with some exceptions for cardiac MRI [96, 97]. Gesture-guided multidimensional editing tools could be helpful to correct AI segmentation results, for example exact tumor volume assessment. Efforts in that arena have been ongoing for more than 15 years [98, 99]. Other AI applications could extract automated vessel diameters and wall thickness or on US signs of fatty liver disease to compare with follow-up studies. Virtual reality devices should allow multidimensional data display and simulation of, for example, interventional procedures. Such AI applications would make radiology reports more precise; enable data tracking, comparison and transparency; and improve patient management and well-being.

### Image display

As mentioned, image display on reporting workstations is based on hanging protocols. It is desirable that AI be trained to guess the individual radiologist’s opinion on any combination of modalities displayed and that the radiologist could shuffle the images with gestures or spoken commands.

### Report generation

Before report generation, AI can search for possible needed relevant resources (textbooks, guidelines, classification schemes) as a background process. Furthermore, based on a standardized AI image description, queries for similar images are performed, which might yield a diagnostic clue. Moreover, AI explores images for patterns (e.g., cancer, occult fractures), which could include available patient data for a diagnosis suggestions. In pediatric radiology this would be especially important for rare diseases, for example congenital malformations or brain diseases caused by metabolic defects.

Moreover, easy sharing with colleagues (local, national, international) for second opinion should be an option and AI could select the best-suited conference partner by analyzing available personal profiles.

At reporting, necessary items could be voice- or gesture-driven, copied from other resources into the report, which should also be capable of holding key images, videos, animations and simulations. All of these tasks would run in the background and in real time, and again information would be displayed intuitively with hovering notes.

Influences on radiology reports could include less description but more precise findings including quantitative data or appropriate scoring of even rare diseases. This achievement would be obtained by the combination of AI-supported information management and human features like content knowledge, creativity, problem-solving capabilities and an excellent exemplary memory — we can remember a case well, even decades later.

### Workplace

An intuitive personalized AI would block unwanted interruptions, store them and sort them on priority for later retrieval.

For some of the mentioned AI applications, the technical basis is already available. The main handicaps are incompatible systems and lack of standardization.

Such AI applications can together be described as a personal AI assistant that serves radiologists at all times.

***This leads the authors to STATEMENT THREE: Undeniably, AI will be an integral part of medicine and especially radiology, but for many reasons the last decision is only for the human radiologist because only his or her education and content knowledge will allow reliable diagnoses.***

However, to use AI properly, a huge collective cross-domain effort is necessary, including: (a) raising awareness; (b) guaranteeing ethical, social and legal aspects of AI; and (c) education spanning all levels of curricula — from students to residents to continuing education. For engineers, human-computer interface redesign as well as strategies for ground truth generation in huge datasets is mandatory.

## References

[1] Ravesloot C, van der Schaaf M, Kruitwagen C (2017). Predictors of knowledge and image interpretation skill development in radiology residents. Radiology.

[2] Nguyen X, Adams S, Hobbs S (2019). Radiologist as lifelong learner: strategies for ongoing education. Acad Radiol.

[3] Neri E, de Souza N, Brady A (2019). What the radiologist should know about artificial intelligence — an ESR white paper. Insights Imaging.

[4] Choy G, Khalilzadeh O, Michalski M (2018). Current applications and future impact of machine learning in radiology. Radiology.

[5] Miyagawa T, Sasaki M, Yamaura A (2020). Intracranial pressure based decision making: prediction of suspected increased intracranial pressure with machine learning. PLoS One.

[6] Longjiang E, Zhao B, Guo Y (2019). Using deep-learning techniques for pulmonary-thoracic segmentations and improvement of pneumonia diagnosis in pediatric chest radiographs. Pediatr Pulmonol.

[7] Zheng Q, Shellikeri S, Huang H (2020). Deep learning measurement of leg length discrepancy in children based on radiographs. Radiology.

[8] Sorantin E (2013). Soft-copy display and reading: what the radiologist should know in the digital era. Pediatr Radiol.

[9] Sorantin E, Weissensteiner S, Hasenburger G, Riccabona M (2013). CT in children — dose protection and general considerations when planning a CT in a child. Eur J Radiol.

[10] Mabotuwana T, Qian Y, Sevenster M (2013). Using image references in radiology reports to support enhanced report-to-image navigation. AMIA Annu Symp Proc.

[11] Klitsch N (2016) How I read imaging studies. Neighborhood Radiologist blog. http://www.neighborhoodradiologist.com/how-i-read-imaging-studies/. Accessed 13 Dec 2020

[12] Bhargavan M, Kaye A, Forman H, Sunshine J (2009). Workload of radiologists in United States in 2006-2007 and trends since 1991-1992. Radiology.

[13] Pitman A, Jones D (2006). Radiologist workloads in teaching hospital departments: measuring the workload. Australas Radiol.

[14] Al Mohammad B, Hillis S, Reed W (2019). Radiologist performance in the detection of lung cancer using CT. Clin Radiol.

[15] European Union (2003) Council directive 2003 88 EC — working time. https://osha.europa.eu/de/legislation/directives/directive-2003-88-ec. Accessed 30 Dec 2020

[16] Krankenanstalten-Arbeitszeitgesetz (2018) [Consolidated federal law: entire legal regulation for the Hospitals Working Hours Act.] https://www.ris.bka.gv.at/GeltendeFassung.wxe?Abfrage=Bundesnormen&Gesetzesnummer=10009051. Accessed 30 Dec 2020

[17] Euler-Chelpin M, Lillholm M, Napolitano G (2018). Screening mammography: benefit of double reading by breast density. Breast Cancer Res Treat.

[18] von Karsa L, Holland R, Broeders M et al (2013) European guidelines for quality assurance in breast cancer screening and diagnosis. Eur Comm Directorate-Gen Health Consumers. 10.2772/13196. Accessed 30 Jun 2021

[19] European Society of Paediatric Radiology (2021) Taskforces. https://www.espr.org/taskforces. Accessed 30 Dec 2020

[20] Thorwarth WT (2008). CPT: an open system that describes all that you do. J Am Coll Radiol.

[21] OTpedia (2021) Hanging protocol. https://otechimg.com/otpedia/entryDetails.cfm?id=153. Accessed 13 Dec 2020

[22] AiDoc Inc. (n.d.) Proven radiology AI. https://www.aidoc.com/. Accessed 04 May 2021

[23] Diagnostic Image Analysis Group, Department of Radiology and Nuclear Medicine at the Radboud University Medical Center (n.d.) AI for radiology — an implementation guide. https://grand-challenge.org/aiforradiology/. Accessed 04 May 2021

[24] Data Science Institute, American College of Radiology (n.d.) Certify-AI. https://www.acrdsi.org/DSI-Services/Certify-AI. Accessed 04 May 2021

[25] Langer S (2002). Radiology speech recognition: workflow, integration, and productivity issues. Curr Probl Diagn Radiol.

[26] Hammana I, Lepanto L, Poder T (2015). Speech recognition in the radiology department: a systematic review. Health Inf Manag.

[27] Wallis A, McCoubrie P (2011). The radiology report — are we getting the message across?. Clin Radiol.

[28] Wang K, Patel J, Vyas B (2017). Use of radiology procedure codes in health care: the need for standardization and structure. Radiographics.

[29] European Society of Radiology (2018). ESR paper on structured reporting in radiology. Insights Imaging.

[30] Turing AM (1950). Computing machinery and intelligence. Mind.

[31] Legg S, Hutter M (2007). A collection of definitions of intelligence. Front Artif Intell Appl.

[32] Copeland BJ (2020) Artificial intelligence. https://www.britannica.com/technology/artificial-intelligence. Accessed 15 Dec 2020

[33] Kotsiantis S, Kanellopoulos D, Pintelas P (2006). Data preprocessing for supervised learning. Int J Comput Sci.

[34] Jamil M, Yang XS (2013). A literature survey of benchmark functions for global optimisation problems. Int J Math Model Numer Optim.

[35] Harrington P, Bleiel J (2012). Manipulating the classifier’s decision with a cost function. Machine learning in action.

[36] Caruana R, Niculescu-Mizil A (2006) An empirical comparison of supervised learning algorithms. In: Cohen W, Moore A (eds) Proceedings of the 23rd International Conference on Machine Learning. ACM, New York, pp 161–168

[37] Ghahramani Z (2004) Unsupervised learning. In: Bousquet O, von Luxburg U, Rätsch G (eds) Advanced lectures on machine learning: ML summer schools 2003, Canberra, Australia, February 2–14, 2003, Tübingen, Germany, August 4–16, 2003. Revised lectures. Springer, Berlin

[38] Zhu XJ (2005) Semi-supervised learning literature survey. Technical report, University of Wisconsin-Madison Department of Computer Sciences. http://digital.library.wisc.edu/1793/60444. Accessed 30 Jun 2021

[39] Beitzel SM, Jensen EC, Frieder O et al (2005) Improving automatic query classification via semi-supervised learning. In: Han J, Wah B, Raghavan V et al (eds) Fifth IEEE International Conference on Data Mining (ICDM’05). IEEE, New York, pp 8–16

[40] Kaelbling LP, Littman ML, Moore AW (1996). Reinforcement learning: a survey. J Artif Intell.

[41] LeCun Y, Bengio Y, Hinton G (2015). Deep learning. Nature.

[42] Hinton GE, Osindero S, Teh YW (2006). A fast learning algorithm for deep belief nets. Neural Comput.

[43] Ruder S (2017) An overview of multi-task learning in deep neural networks. http://arxiv.org/abs/1706.05098

[44] O’Shea K, Nash R (2015) An introduction to convolutional neural networks. http://arxiv.org/abs/1511.08458

[45] Mikolov T, Kombrink S, Burget L et al (2011) Extensions of recurrent neural network language model. In: 2011 IEEE International Conference on Acoustics, Speech and Signal Processing (ICASSP). IEEE, New York, pp 5528–5531

[46] Hatcher WG, Yu W (2018). A survey of deep learning: platforms, applications and emerging research trends. IEEE Access.

[47] Yip S, Liu Y, Parmar C (2017). Associations between radiologist-defined semantic and automatically computed radiomic features in non-small cell lung cancer. Sci Rep.

[48] Thibault G, Angulo J, Meyer F (2014). Advanced statistical matrices for texture characterization: application to cell classification. IEEE Trans Biomed Eng.

[49] Galloway MM (1975). Texture analysis using gray level run lengths. Comput Gr Image Process.

[50] Fujita H (2020). AI-based computer-aided diagnosis (AI-CAD): the latest review to read first. Radiol Phys Technol.

[51] Giger M, Karssemeijer N, Schnabel J (2013). Breast image analysis for risk assessment, detection, diagnosis, and treatment of cancer. Annu Rev Biomed Eng.

[52] Hržić F, Štajduhar I, Tschauner S (2019). Local-entropy based approach for X-ray image segmentation and fracture detection. Entropy.

[53] Noble WS (2006). What is a support vector machine?. Nat Biotechnol.

[54] Chen KC, Chen CYC (2011). Stroke prevention by traditional Chinese medicine? A genetic algorithm, support vector machine and molecular dynamics approach. Soft Matter.

[55] Battineni G, Chintalapudi N, Amenta F (2019). Machine learning in medicine: performance calculation of dementia prediction by support vector machines (SVM). Inform Med Unlocked.

[56] Lambin P, Rios Velazquez E, Leijenaar R (2012). Radiomics: extracting more information from medical images using advanced feature analysis. Eur J Cancer.

[57] Hosny A, Parmar C, Quackenbush J (2018). Artificial intelligence in radiology. Nat Rev Cancer.

[58] Lorencin I, Andjelic N, Spanjol J, Car Z (2020). Using multi-layer perceptron with Laplacian edge detector for bladder cancer diagnosis. Artif Intell Med.

[59] Ronneberger O, Fischer P, Brox T (2015) U-Net: convolutional networks for biomedical image segmentation. http://arxiv.org/abs/1505.04597

[60] Bi WL, Hosny A, Schabath M (2019). Artificial intelligence in cancer imaging: clinical challenges and applications. CA Cancer J Clin.

[61] Tezcan KC, Baumgartner CF, Luechinger R (2019). MR image reconstruction using deep density priors. IEEE Trans Med Imaging.

[62] Wolterink JM, Dinkla AM, Savenije MH et al (2017) Deep MR to CT synthesis using unpaired data. In: Tsaftaris SA, Gooya A, Frangi AF, Prince JL (eds) International Workshop on Simulation and Synthesis in Medical Imaging. Springer, New York, pp 14–23

[63] Pu Y, Gan Z, Henao R (2016). Variational autoencoder for deep learning of images, labels and captions. Adv Neural Inf Process Syst.

[64] Badretale S, Shaker F, Babyn P, Alirezaie J (2017) Deep convolutional approach for low-dose CT image noise reduction. In: 2017 24th National and 2nd International Iranian Conference on Biomedical Engineering (ICBME). IEEE, New York, pp 1–5

[65] Chen H, Zhang Y, Zhang W (2017). Low-dose CT via convolutional neural network. Biomed Optics Express.

[66] Nadkarni PM, Ohno-Machado L, Chapman WW (2011). Natural language processing: an introduction. J Am Med Inform Assoc.

[67] Lee JG, Jun S, Cho YW (2017). Deep learning in medical imaging: general overview. Korean J Radiol.

[68] Lugo-Fagundo C, Vogelstein B, Yuille A, Fishman EK (2018). Deep learning in radiology: now the real work begins. J Am Coll Radiol.

[69] Schneeberger D, Stoeger K, Holzinger A (2020) The European legal framework for medical AI. In: Proceedings of the International Cross-Domain Conference for Machine Learning and Knowledge Extraction, Fourth IFIP TC 5, TC 12, WG 8.4, WG 8.9, WG 12.9 International Cross-Domain Conference, CD-MAKE 2020. Springer, Cambridge, pp 209–226

[70] Holzinger A, Kieseberg P, Weippl E, Tjoa AM (2018). Current advances, trends and challenges of machine learning and knowledge extraction: from machine learning to explainable AI. Springer lecture notes in computer science LNCS 11015.

[71] Montavon G, Samek W, Mueller KR (2018). Methods for interpreting and understanding deep neural networks. Digit Sign Process.

[72] Holzinger A, Langs G, Denk H (2019). Causability and explainability of artificial intelligence in medicine. Wiley Interdiscip Rev Data Min Knowl Discov.

[73] Holzinger A, Carrington A, Mueller H (2020). Measuring the quality of explanations: the system causability scale (SCS): comparing human and machine explanations. Kunstliche Intell.

[74] Holzinger A, Errath M, Searle G (2005). From extreme programming and usability engineering to extreme usability in software engineering education. 29th International Annual IEEE Computer Software and Applications Conference (IEEE COMPSAC 2005).

[75] Holzinger A, Plass M, Kickmeier-Rust M (2019). Interactive machine learning: experimental evidence for the human in the algorithmic loop. Appl Intell.

[76] Choi J, Kim S, Kang B (2013). Mammographic artifacts on full-field digital mammography. J Digit Imaging.

[77] Pinckaers H, van Ginneken B, Litjens G (2019) Streaming convolutional neural networks for end-to-end learning with multi-megapixel images. arXiv:1911.04432 10.1109/TPAMI.2020.3019563 32845835

[78] Mackin D, Fave X, Zhang L (2015). Measuring CT scanner variability of radiomics features. Investig Radiol.

[79] Armato SG, McLennan G, Bidaut L (2011). The lung image database consortium (LIDC) and image database resource initiative (IDRI): a completed reference database of lung nodules on CT scans. Med Phys.

[80] Wang X, Peng Y, Lu L et al (2017) ChestX-ray8: hospital-scale chest X-ray database and benchmarks on weakly-supervised classification and localization of common thorax diseases. In: Proceedings of the IEEE Conference on Computer Vision and Pattern Recognition. IEEE, Los Alamitos, pp 2097–2106

[81] Irvin J, Rajpurkar P, Ko M (2019). CheXpert: a large chest radiograph dataset with uncertainty labels and expert comparison. Proc AAAI Conf Artif Intell.

[82] Wilson AG, Gilboa E, Nehorai A, Cunningham JP, Ghahramani Z, Welling M, Cortes C (2014). Fast kernel learning for multidimensional pattern extrapolation. Advances in neural information processing systems (NIPS 2014).

[83] Auer P, Long PM, Maass W, Woeginger GJ (1995). On the complexity of function learning. Mach Learn.

[84] Wolpert DM, Ghahramani Z, Jordan MI (1995). An internal model for sensorimotor integration. Science.

[85] Lucas CG, Griffiths TL, Williams JJ, Kalish ML (2015). A rational model of function learning. Psychon Bull Rev.

[86] Knill DC, Pouget A (2004). The Bayesian brain: the role of uncertainty in neural coding and computation. Trends Neurosci.

[87] Tenenbaum JB, Griffiths TL, Kemp C (2006). Theory-based Bayesian models of inductive learning and reasoning. Trends Cogn Sci.

[88] Holzinger A (2020). Explainable AI and multi-modal causability in medicine. Wiley i-com J Interact Media.

[89] Holzinger A, Malle B, Saranti A, Pfeifer B (2021). Towards multi-modal causability with graph neural networks enabling information fusion for explainable AI. Inform Fusion.

[90] Zhou Y, Teomete U, Dandin O (2016). Computer-aided detection (CADx) for plastic deformation fractures in pediatric forearm. Comput Biol Med.

[91] Choi JW, Cho YJ, Lee S (2020). Using a dual-input convolutional neural network for automated detection of pediatric supracondylar fracture on conventional radiography. Investig Radiol.

[92] Oakden-Rayner L (2020). Exploring large-scale public medical image datasets. Acad Radiol.

[93] European Society of Radiology (2019) ESR iGuide. https://www.myesr.org/esriguide. Accessed 30 Dec 2020

[94] GE Healthcare (2021) TrueFidelity: how the best see better. https://www.gehealthcare.com/products/truefidelity. Accessed 07 May 2021

[95] Hsieh J, Liu E, Nett B et al (2019) A new era of image reconstruction: TrueFidelity: technical white paper on deep learning image reconstruction. https://www.gehealthcare.com/-/jssmedia/040dd213fa89463287155151fdb01922.pdf. Accessed 07 May 2021

[96] Circle Cardiovascular Imaging (n.d.) Circle Cardiovascular Imaging deep learning story. https://www.circlecvi.com/cvi42/cardiac-mri/deep-learning/. Accessed 30 Dec 2020

[97] Shirakawa T (2020) A.I.Segmentation. https://compositecreatures.jimdofree.com/a-i-segmentation/. Accessed 30 Dec 2020

[98] Reitinger B, Bornik A, Beichel R, Schmalstieg D (2006). Liver surgery planning using virtual reality. IEEE Comput Graph Appl.

[99] Beichel R, Bornik A, Bauer C, Sorantin E (2012). Liver segmentation in contrast enhanced CT data using graph cuts and interactive 3D segmentation refinement methods. Med Phys.

